# Proteostasis in the Male and Female Germline: A New Outlook on the Maintenance of Reproductive Health

**DOI:** 10.3389/fcell.2021.660626

**Published:** 2021-04-16

**Authors:** Shenae L. Cafe, Brett Nixon, Heath Ecroyd, Jacinta H. Martin, David A. Skerrett-Byrne, Elizabeth G. Bromfield

**Affiliations:** ^1^Priority Research Centre for Reproductive Science, Faculty of Science, The University of Newcastle, Callaghan, NSW, Australia; ^2^Molecular Horizons, School of Chemistry and Molecular Bioscience, University of Wollongong, Wollongong, NSW, Australia; ^3^Illawarra Health and Medical Research Institute, Wollongong, NSW, Australia; ^4^Department of Human Genetics, McGill University Health Centre Research Institute, Montreal, QC, Canada; ^5^Department of Biochemistry and Cell Biology, Faculty of Veterinary Medicine, Utrecht University, Utrecht, Netherlands

**Keywords:** protein homeostasis, reproductive aging, oxidative stress, chaperone, germ cell, oocyte, sirtuin, spermatozoa

## Abstract

For fully differentiated, long lived cells the maintenance of protein homeostasis (proteostasis) becomes a crucial determinant of cellular function and viability. Neurons are the most well-known example of this phenomenon where the majority of these cells must survive the entire course of life. However, male and female germ cells are also uniquely dependent on the maintenance of proteostasis to achieve successful fertilization. Oocytes, also long-lived cells, are subjected to prolonged periods of arrest and are largely reliant on the translation of stored mRNAs, accumulated during the growth period, to support meiotic maturation and subsequent embryogenesis. Conversely, sperm cells, while relatively ephemeral, are completely reliant on proteostasis due to the absence of both transcription and translation. Despite these remarkable, cell-specific features there has been little focus on understanding protein homeostasis in reproductive cells and how/whether proteostasis is “reset” during embryogenesis. Here, we seek to capture the momentum of this growing field by highlighting novel findings regarding germline proteostasis and how this knowledge can be used to promote reproductive health. In this review we capture proteostasis in the context of both somatic cell and germline aging and discuss the influence of oxidative stress on protein function. In particular, we highlight the contributions of proteostasis changes to oocyte aging and encourage a focus in this area that may complement the extensive analyses of DNA damage and aneuploidy that have long occupied the oocyte aging field. Moreover, we discuss the influence of common non-enzymatic protein modifications on the stability of proteins in the male germline, how these changes affect sperm function, and how they may be prevented to preserve fertility. Through this review we aim to bring to light a new trajectory for our field and highlight the potential to harness the germ cell’s natural proteostasis mechanisms to improve reproductive health. This manuscript will be of interest to those in the fields of proteostasis, aging, male and female gamete reproductive biology, embryogenesis, and life course health.

## Introduction

A functional proteome is essential for the survival of cells and organisms, resulting in a considerable investment of energy into the maintenance of cellular protein homeostasis (proteostasis). As energy demands change in response to the environment, such as during aging and oxidative stress, proteostasis can be compromised leading to a harmful accumulation of damaged or misfolded proteins and the induction of degenerative diseases such as Alzheimer’s disease (AD) and Amyotrophic lateral sclerosis (ALS). For the male and female germline, the regulation of proteostasis can be particularly challenging as the male gamete experiences prolonged periods in the relative absence of transcription and translation while the female gamete is similarly transcriptionally inactive prior to fertilization and embryonic genome activation. Consequently, maintaining a functional proteome becomes heavily reliant upon proteostasis machinery, the cellular environment and post-translational protein regulation.

Distinctively, in the case of the gametes, the stakes are extremely high as the maintenance of proteostasis throughout the life of germ cells is critical to ensure the fitness of the next generation. In recent years, a focus on characterizing the proteomes of germ cells has begun to reveal the importance of proteostasis both to cellular and organismal development, as well as for fertilization. Herein, we provide an important update on the contribution of proteostasis disruption to male and female infertility and highlight key areas for future research through the specific lenses of reproductive aging and oxidative stress; two of the most prevalent elements that compromise germ cell function and contribute to infertility in humans. Moreover, we draw on relevant discoveries in somatic cells and model organisms to propose a new trajectory for the field of mammalian reproduction that may lead to better regulation of proteostasis and the improvement of male and female reproductive health.

## The Proteostasis Network in Health and Disease

Proteostasis describes the homeostatic relationship between protein production, assembly, and degradation. It is thought that up to 30% of newly synthesized proteins are misfolded as a consequence of the complex intracellular milieu, where macromolecular crowding poses as a major challenge to the delicate state of equilibrium between the native and non-native conformations of proteins (Grune et al., 2004; Houck et al., 2012). Following translation, most nascent polypeptides must correctly fold into their three-dimensional structures prior to fulfilling their functional roles. Disruptions in this process potentiates protein aggregation, in turn, requiring cells to engage a consortium of defense mechanisms in order to mitigate the impact of misfolded protein species (Dobson, 2004; Schaur et al., 2015; Radwan et al., 2017). The proteostasis network (PN) includes elements capable of modulating protein synthesis/translation, trafficking, folding, secretion and degradation; and can be altered transiently or permanently in response to stress and other compounding factors (Koga et al., 2011; Kim et al., 2013).

Protein aggregation can be triggered by numerous cellular challenges including oxidative stress-induced unfolding/misfolding, a compromised proteostasis network, or age-dependent decline in protein folding machinery, diminished quality control, as well as mutations that increase the propensity of a protein to aggregate (Stefani, 2004). Different forms of aggregates can arise from the same protein due to exposure to diverse stress conditions. The term aggregate has broad connotations, referring to composites of misfolded proteins that compromise cellular function (Hohn et al., 2013; Mattoo and Goloubinoff, 2014; Radwan et al., 2017). Similarly, whilst the term amyloid refers to a type of insoluble protein aggregates, these are dominated by β-sheet secondary structures and a fibrillar morphology and often found within inclusion bodies (Stefani, 2004). Concordantly, protein aggregation, and the accumulation of the toxic protein species as a result of this process, is often indicative of a decline in proteostasis (Radwan et al., 2017). The unchecked propagation of protein aggregates often accompanies the onset of severe disease states (Chiti and Dobson, 2006), with notable examples including AD, ALS, type 2 diabetes, and the spongiform encephalopathies (e.g., mad cow disease); all of which are progressive disorders associated with high rates of morbidity and mortality (Rambaran and Serpell, 2008).

### Key Components of the Proteostasis Network Relevant to This Review

**FIGURE 1:**
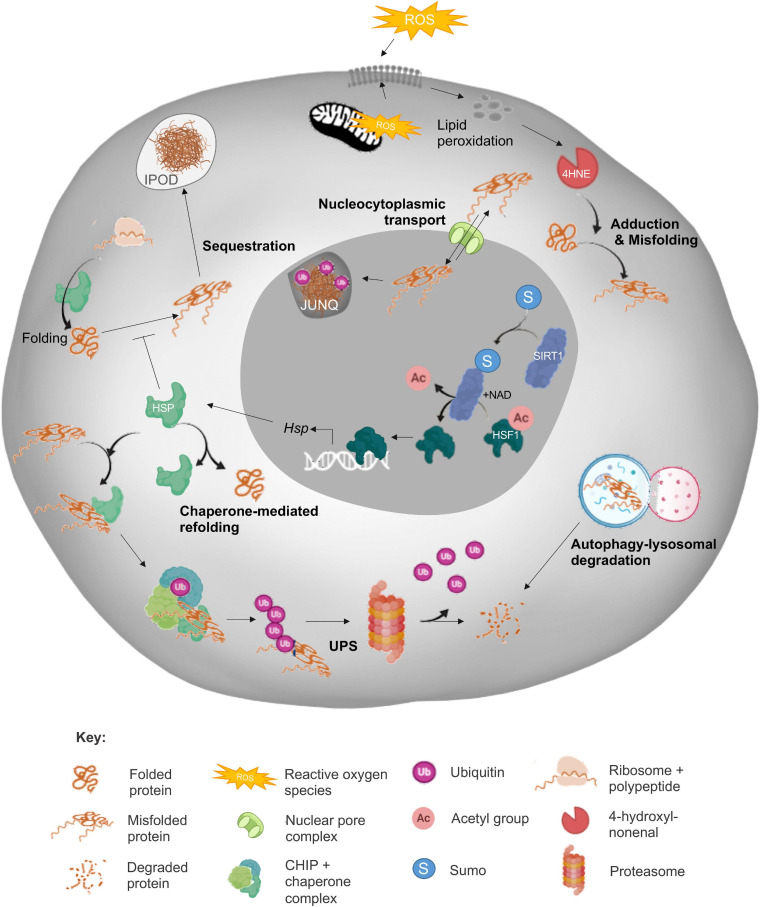
Summary of the cellular proteostasis network. The fate of misfolded proteins arising from oxidative stress (ROS)-induced cascades varies depending on the nature of the protein and extent of damage. As depicted, adduction by reactive carbonyl species (RCS) such as 4-hydroxynonenal (4HNE) can elicit protein misfolding and necessitate the engagement of a panoply of cellular defense mechanisms designed to prevent propagation of protein damage and/or directly eliminate misfolded proteins. Among these mechanisms, the sensing of redox state and subsequent induction of heat shock response (HSR) by sirtuins (SIRT) leads to the upregulation of heat shock protein (Hsp) expression. The HSPs, or chaperone proteins are capable of directing misfolded targets toward pathways of protein refolding or targeted degradation via the ubiquitin-proteasomal pathway (UPS). As a more direct, but less selective route of degradation, misfolded proteins can also be recycled via autophagy/lysosomal machinery. Alternatively, misfolded proteins can be compartmentalized into insoluble protein deposits (IPOD) or sequestered into juxtanuclear quality control compartments (JUNQs) via shuttling between the cytoplasm and nucleus directed by nucleocytoplasmic transport machinery. Elements of this figure were made in BioRender.

In view of the deleterious impact of protein aggregation, it is perhaps not surprising that somatic cells are generally endowed with a sophisticated suite of cellular surveillance and defense mechanisms designed to detect, counter and/or repair protein aggregate formation. Such mechanisms include the synergistic action of the heat shock response (HSR), unfolded protein response (UPR^*ER*^ and UPR^*mt*^), ubiquitin-proteasome system (UPS) and autophagic-lysosomal pathways; all of which combine to play crucial roles in the maintenance of cellular homeostasis (Figure 1). Whilst each of these mechanisms are undoubtedly important, here we focus only on those elements of the PN summarized in Figure 1 in order to provide context to proteostasis mechanisms as they relate to male and female fertility. For a comprehensive review of all proteostasis elements and mechanisms see Labbadia and Morimoto, 2015 and Klaips et al., 2018.

#### Protein Degradation

As a primary line of defense against aggregation-induced cellular toxicity, the proteasome complex is responsible for the catalytic degradation of irreversibly damaged and/or dysfunctional proteins and the recycling of their components. Proteins destined for proteasomal degradation are tagged with polyubiquitin chain(s) consisting of four or more ubiquitin moieties, via the action of one of the more than 600 ubiquitin ligases represented in the human genome (Iconomou and Saunders, 2016; Zheng and Shabek, 2017). The assembly of specific proteasome subunits directs the formation of the 20S proteasome, 26S proteasome (consisting of the 20S core plus 19S regulatory cap) and the immunoproteasome (20S in combination with an 11S regulatory subunit), among other tissue specific forms (Grune et al., 2004; Hohn et al., 2013). In somatic cells, proteasome activity is up-regulated in response to moderate levels of oxidative stress and thereafter it fulfils a key role in the resolution of dysfunctional and misfolded proteins (Ding et al., 2003; Pickering et al., 2010). In contrast, exposure to chronic and/or intense oxidative insults can lead to proteasome inhibition and in extreme situations, complete disassembly (Davies, 2001; Ferrington and Kapphahn, 2004; Aiken et al., 2011). Proteasome activity is also decreased in multiple organs with increased age and, in model species such as *Drosophila*, these insults have contributed to defective 20S and 19S association leading to compromised 26S proteasome assembly (Tonoki et al., 2009). Alternatively, proteasomal dysfunction may also be caused by post-translational modifications (PTMs) and the extent of substrate aggregation (Keller et al., 2000; Im and Chung, 2016), with heavily cross-linked proteins demonstrated to “clog” the proteasome and even cause proteasomal inhibition (Hohn et al., 2013).

Crosstalk between the UPS and the autophagy-lysosomal system is proposed to occur through the conformation of ubiquitin chains as well as the activity of the autophagy receptor p62 (Kocaturk and Gozuacik, 2018). As a compensatory, or alternative, mechanism to the UPS, autophagy is a catabolic process responsible for the degradation of superfluous and dysfunctional organelles, long-lived proteins and protein aggregate structures and can be categorized into three distinct subtypes, macroautophagy, chaperone-mediated autophagy, and mitophagy (Lilienbaum, 2013; Peters et al., 2020). Macroautophagy (hereafter referred to as autophagy) is characterized by the formation of double membrane bound structures, autophagosomes, and is regulated by mammalian target of rapamycin (mTOR). Initially described as a non-selective waste disposal pathway, selective roles for autophagy have since been reported with regards to the removal of protein aggregates or inclusions, a process termed aggrephagy (Mancias and Kimmelman, 2016). As a clearance mechanism for protein aggregates, aggrephagy has been investigated using multimerized fluorescent particles which track the interaction of clusters (mimicking aggregates) with ubiquitin prior to the recruitment of p62 and microtubule-associated protein 1A/1B-light chain 3 (LC3), preceding lysosomal degradation (Janssen et al., 2018). The selective nature of aggrephagy rests with a combination of molecular chaperones such as BAG family molecular chaperone regulator 3 (BAG3) (Gamerdinger et al., 2011; Lamark and Johansen, 2012; Klimek et al., 2017) and autophagy receptors such as p62 and TAX1BP1 (Bjorkoy et al., 2005; Pankiv et al., 2007; Sarraf et al., 2019), the latter of which links cargo to autophagosomes.

#### Molecular Chaperones

The proteasome and lysosome are by no means the only components of the PN that are dysregulated in response to aging and oxidative stress. Indeed, it has been shown that nearly 50% of molecular chaperones expressed in the human brain are differentially regulated equivalently upon aging or in response to the development of neurodegenerative conditions (Smith et al., 2015). Interestingly, 70% of the human “chaperome,” consisting of >332 genes responsible for chaperone and co-chaperone production (Brehme et al., 2014), is dysregulated in opposing directions between cancer and neurodegeneration, corroborating the notion that the collapse of the proteostasis network is responsible for a wide range of pathogenic conditions (Hadizadeh Esfahani et al., 2018).

The proteostasis network is modulated by an armada of chaperones functioning as “holdases” and “foldases” (Hipp et al., 2019). In their capacity as holdases, chaperones temporarily prevent protein aggregation by binding to exposed hydrophobic regions of proteins that would otherwise trigger aggregation (Mattoo and Goloubinoff, 2014; Diaz-Villanueva et al., 2015; Hipp et al., 2019). Alternatively, those chaperones with foldase activity facilitate protein stabilization, folding, disaggregation and refolding. Although some chaperones are constitutively expressed, the ability of some chaperones to be induced by stress enables cells to adapt to changing environmental conditions and intracellular signals (Hohn et al., 2013). The chaperone machinery expressed in mammals, are broadly divided into five main families based on molecular weight, namely the small heat shock proteins (HSPs), HSP40s, HSP60s/CCT chaperonins, HSP70s/110 and HSP90s (Hartl et al., 2011; Dun et al., 2012).

As a reflection of its importance as a protein stabilizer within the PN (Hohn et al., 2013), HSP90 proteins account for approximately 1–2% of all proteins in eukaryotic cells (Niforou et al., 2014). The members belonging to the HSP90 protein family are highly conserved molecules with an approximate molecular weight of 90 kDa. The Human HSP90 family includes five members with the overall structure of HSP90 homologs comprising three conserved domains; N-terminal domain, C-terminal domain and middle domain (Hoter et al., 2018). HSP90 proteins commonly work in concert with HSP70 (Mayer and Bukau, 2005; Labbadia et al., 2012; Radwan et al., 2017) to recruit the C-terminus of HSC70 interacting protein (CHIP), which in turn ubiquitinates misfolded proteins (Figure 1) to direct them away from refolding, and toward proteasomal degradation (Shang and Taylor, 2011; Houck et al., 2012; Hipp et al., 2019). Recent work from our laboratory has implicated HSP90 in the mitigation of protein aggregate accumulation in developing male germ cells; such that selective HSP90 inhibition resulted in a significant increase in protein aggregates (Cafe et al., 2020). Such findings imply that the action of the HSP90 chaperone family in aggregate mitigation may be conserved in germ cells and somatic cells. Similarly, the HSP70 chaperone family also fulfils ancillary roles in protein transport, folding, degradation, dissolution of protein complexes and control of regulatory proteins (Daugaard et al., 2007). By way of example, an 8-fold increase in HSP70 abundance has been recorded in neuronal cells harboring mutant huntingtin protein [mHtt; an amyloidogenic mutant that drives the progression of Huntington’s disease (HD)] (Tagawa et al., 2007). Such a result may arise as a consequence of the sequestration of HSP70, and its co-chaperone HSP40, into aggregates in HD, thus reducing their availability to participate in refolding (Simoes-Pires et al., 2013). The importance of the HSP40/70 activity is demonstrated by the ability of this complex to reduce polyglutamine (polyQ) formation, and superoxide dismutase 1 (SOD1; the major protein involved in ALS pathogenesis) aggregation (Wacker et al., 2004; Smith et al., 2015). Additionally, the intercellular transmission of HSP40/70, via exosomes, can fortify the PN defenses of cells containing aggregated proteins (Takeuchi et al., 2015).

The small heat shock protein family (sHSPs) enables cells to respond to stress by stabilizing aggregation-prone proteins. Members such as HSP27 can target damaged proteins for UPS-mediated degradation, while HSP22 promotes autophagy-mediated degradation of targets (Niforou et al., 2014). sHSPs prevent aggregation of SOD1 (Yerbury et al., 2013), and promote autophagic clearance through the interaction of HSP22 with CHIP/HSC70/BAG3 (Crippa et al., 2010). As such, HSP22 and HSP20 are elevated in response to AD, HD, and ALS, and HSP27 is found in tangles of hyperphosphorylated tau in AD (Treweek et al., 2015).

### Compartmentalization and Partitioning

A widely conserved mechanism to combat the toxicity of protein aggregates is through the compartmentalization and/or sequestration of misfolded proteins within cells. In several studies it has been shown that cytoplasmic aggregates exhibit higher toxicity than their nuclear counterparts, possibly due to the sequestration of aggregates into intranuclear inclusions (Woerner et al., 2016) and interactions with compartment specific chaperones (Da Cruz and Cleveland, 2016). The three main examples of this compartmentalization include the formation of aggresomes at the microtubule organizing center (Kawaguchi et al., 2003; Simoes-Pires et al., 2013), the sequestration of ubiquitinated misfolded proteins into juxtanuclear quality control compartments (JUNQs) and the direction of terminally aggregated proteins to insoluble protein deposits (IPODs; Figure 1; Kaganovich et al., 2008; Radwan et al., 2017) for resolution via autophagy (Hohn et al., 2013). Furthermore, recent research in this field has implicated the nucleocytoplasmic transport machinery in the regulation of proteostasis in somatic cells (Shibata and Morimoto, 2014).

The nuclear envelope enforces spatial separation of the nucleus and cytosol of eukaryotic cells and consequently the segregation of genetic material, transcriptional and translational machinery, and metabolic systems. As such, there is a requirement for transport machinery to recognize and regulate the movement of molecular components across the nuclear envelope (Pemberton and Paschal, 2005; Lange et al., 2007). The intracellular distribution, and hence the mislocalization of proteins, appears to be key to their toxicity (Ferrigno and Silver, 2000; Barmada et al., 2010). Accordingly, mutations in the nuclear localization sequence (NLS) of misfolded proteins (the sequence that targets them for nuclear import) increases their toxicity (Woerner et al., 2016), with their aberrant interactions with the nuclear pore contributing to protein conformational disorders including ALS and tauopathies (Eftekharzadeh et al., 2018). Additionally, the mislocalization of nuclear import machinery (KPNA2 and B1), along with nuclear pore components, has been found in the brains and spinal tissue of ALS patients (Kim and Taylor, 2017). Moreover, cytoplasmic aggregates have the ability to disrupt the transport of other biomolecules through the nuclear pore (Woerner et al., 2016; Radwan et al., 2017), further perpetuating proteostatic imbalance. Such defects can be perpetuated from the host cell onto daughter cells, with protein aggregate inclusions (IPOD and JUNQ) known to be asymmetrically inherited into daughter cells at the cost of mitosis speed and lifespan of the daughter cell (Rujano et al., 2006). Whilst a myriad of karyopherin proteins have also been identified in the male (Loveland et al., 2015) and female germ lines (Mihalas et al., 2015), we are only just beginning to understand the role they may play in mitigating the risk posed by misfolded proteins in these cells (Cafe et al., 2020).

### Post-translational Modifications

Owing to their innate ability to alter protein structure and function, PTMs can modulate and disrupt biological processes associated with cellular longevity, reproduction and neurodegeneration (Sambataro and Pennuto, 2017; Santos and Lindner, 2017). There are hundreds of PTMs that proteins can acquire during the life-course of a cell, ultimately giving rise to a dynamic proteome that is vastly more complex than the genome would suggest (Kulkarni and Uversky, 2018). Occurring via both enzymatic and non-enzymatic means, common forms of PTMs include phosphorylation, glycosylation, acetylation, methylation, sumoylation, palmitoylation, biotinylation, ubiquitination, nitration, chlorination, and oxidation/reduction. Illustrative of the extent of PTMs, it has been proposed that as many as 80–90% of proteins may be acetylated in eukaryotic organisms (Arnesen et al., 2009), while approximately 30% are subject to phosphorylation (Cohen, 2000).

Unlike physiological PTMs, carbonylation reactions are generally held to be detrimental and capable of leading to protein misfolding, crosslinking and eventually aggregation (Petersen and Doorn, 2004), that is archetypal of many disease states (Bizzozero et al., 2005; Liu et al., 2005; Reed et al., 2009). Protein carbonylation refers to chemical modification of the primary structure of a protein via the addition of reactive carbonyl species (RCS), a prime example of which is 4-hydroxynonenal (4HNE) adduction. 4HNE exhibits dose-dependent effects on cellular function with lower doses increasing proteasome activity and higher doses being incongruous with cellular function, leading to protein aggregation, proteasomal inhibition and eventually cell death (Aiken et al., 2011; Bromfield et al., 2015, 2017; Nixon et al., 2019a). Elevated concentrations of 4HNE have also been detected in association with various neurodegenerative diseases and causally linked to disease pathologies via adduction and dysregulation of the cellular proteome (Yoritaka et al., 1996; Markesbery and Lovell, 1998; Schaur et al., 2015). Illustrative of this is the tau protein, which is known to be modified by 4HNE resulting in the stabilization of neurofibrillary tangles (Liu et al., 2005). Additionally, as a potential contributor to the propagation of protein aggregation, 4HNE treatment of neurons results in a 2.5-fold increase in the packaging of α-synuclein (the aggregation of which is responsible for Parkinson’s disease) oligomers/fibrils into extracellular vesicles, which can be internalized by untreated neurons, thus disseminating aggregates and enhancing toxicity (Zhang et al., 2018). As discussed in the sections below, 4HNE treatment also elicits pervasive impacts in both the male and female germlines.

Aside from carbonylation, ubiquitination, nitration and phosphorylation modifications have also been linked to the aggregation of proteins such as α-synuclein with downstream consequences including neurotoxicity (Santos and Lindner, 2017). Additionally, as ubiquitination often competes for the same residues as acetylation, this can accentuate protein aggregate accumulation. Indeed, it has been shown that insufficient levels of SIRT1, can preclude proteasome-mediated degradation of substrates since the acetylation of lysine residues effectively prevents access of ubiquitin ligases (Min et al., 2013). In a similar context, hyperphosphorylation of tau can dramatically increase aggregate formation and is associated with tau tangles in AD (Fujiwara et al., 2002; Ren et al., 2014). In addition to the ability of PTMs to modify the activity of individual proteins, at times promoting their aggregation, PTMs have also been shown to decrease proteasome activity through direct adduction. Both the carbonylation and oxidative modification of proteasome subunits has been reported (Ishii et al., 2005; Hohn and Grune, 2014), resulting in destabilization of the proteasome complex and a commensurate inhibition of its catalytic activity. Such modifications reduce proteolytic degradation of misfolded or damaged proteins, leaving them instead to form aggregates (Grune et al., 2004).

Considering the mature sperm cell is transcriptionally and translationally quiescent, it must rely on the cumulative efforts of PN components to maintain the fidelity of intrinsic proteins during post-testicular maturation (Maciel et al., 2019). Fittingly, PTMs are crucial for the acquisition of sperm functional competence, playing key roles in capacitation-associated signaling events that underpin hyperactivated motility and oocyte interactions (Aitken and Nixon, 2013; Nixon and Bromfield, 2018). Whilst they are crucial for normal function, PTMs are interlinked with diverse disease states, extending beyond neurodegeneration to include infertility. Highlighting this, excessive levels of reactive nitrogen species (RNS)-induced modifications have been documented in the defective spermatozoa of asthenozoospermic patients (Lefievre et al., 2007; Martinez-Heredia et al., 2008). Additionally, dysregulated glycosylation, acetylation, ubiquitination, and SUMOylation are linked to abnormal sperm morphology and impaired fertilization and embryo development (Samanta et al., 2016; Maciel et al., 2019). These protein modifications represent important examples of the parallels that exist between germ and somatic cell proteostasis. Indeed, a range of features associated with neurodegenerative conditions, including dysregulation of the PN, are common to the pathologies of both male and female infertility (Supplementary Table 1). This information is critical to our understanding of how we may manipulate the PN to prevent fertility loss. Accordingly, in the following sections we consider to the contribution of proteostasis to male and female germ cell development and fertility.

## Maintaining Proteostasis During Male Germ Cell Development

Advanced paternal age has been linked to declines in semen quality with a reduction in volume and sperm motility (Frattarelli et al., 2008), increased morphological abnormalities [most pronounced in men over 50; (Kidd et al., 2001)], increased time to pregnancy (Ford et al., 2000) and an increased mutational load due to DNA damage (Petersen et al., 2018; Yatsenko and Turek, 2018; Pino et al., 2020). Additionally, evidence is mounting in support of a link between advanced paternal age and the incidence of developmental abnormalities in offspring including autism and schizophrenia (Drevet and Aitken, 2020), but the etiologies underpinning reductions in semen quality remain largely unresolved. Notwithstanding this increasing evidence that paternal age does pose concerns in terms of both male fertility and offspring health, the role that proteostasis disruption may play in these issues remains uncharacterized. Despite this, in the context of sperm production and function, protein homeostasis and the PN network are known to be critical for successful sperm production and maturation (Dun et al., 2012) and in the survival and function of mature spermatozoa that carry out fertilization in the relative absence of transcription and translation (Bromfield et al., 2015). Moreover, it is well known that the mature spermatozoon is highly sensitive to oxidative protein damage due to a lack of cytoplasmic antioxidants and functional detoxifying machinery (Drevet and Aitken, 2020). Thus, irreversible protein damage, such as that elicited by RCS and other lipid peroxidation products, can rapidly affect sperm cell motility and viability (Bromfield et al., 2019). Given this, it is not surprising that oxidative stress is one of the major contributing factors to clinical male infertility.

Herein, we discuss what is known regarding protein homeostasis across the specific stages of sperm cell development and how the PN network relates to male fertility, especially in the context of oxidative cellular damage.

### Testicular Germ Cells

**FIGURE 2:**
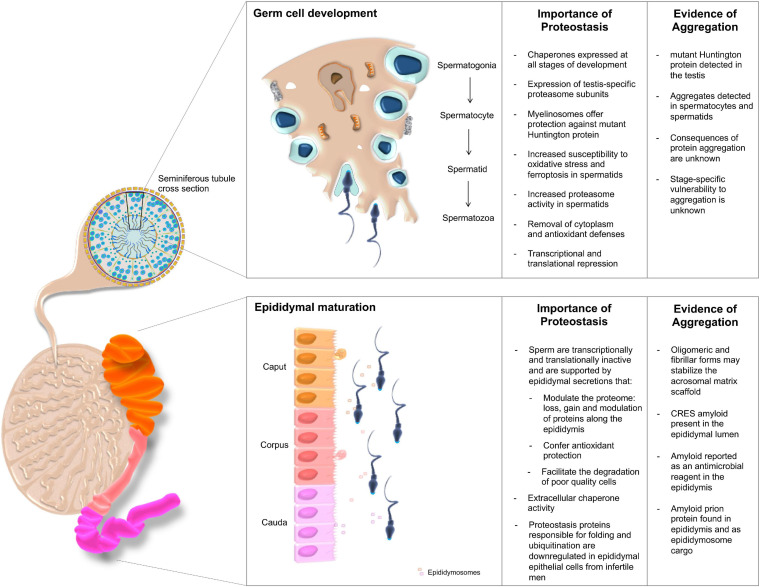
The importance of proteostasis throughout male germ cell development. Male germ cells are produced through successive waves of spermatogenesis occurring within the seminiferous tubules of the testes, yet acquire functional competence during their subsequent transport through the epididymis. The integrity of both maturational phases is intimately regulated by complex proteostasis networks, which work in concert to secure the continuous production of high quality spermatozoa. Despite these quality control measures, the formation of protein aggregates has been documented at various sites within the male reproductive tract. Although their origins remain largely enigmatic, such deposits have been ascribed both pathological and functional roles.

In mammals, the establishment of a self-populating stem cell niche in the testis occurs during early embryonic development and underpins the ability to produce gametes at sexual maturation. The male gametes, spermatozoa, are produced through a synchronized and highly complex differentiation pathway known as spermatogenesis (Figure 2). The stringent regulation of spermatogenesis is enabled by the compartmental organization of the testis; tight junctions between Sertoli cells form a blood-testis barrier that gives rise to an immune-privileged intratubular environment, which is maintained with support from the Sertoli and peritubular myoid cells as well as Leydig cells residing in the interstitium (Hermo et al., 2010; Garcia and Hofmann, 2015). All of these factors are required to act in concert with one another and in response to inter- and intra-cellular signals, with any disruptions to the process of spermatogenesis having the potential to result in subfertility.

Similar to somatic cells, developing male germ cells are protected through the action of the UPS and formation of the spermatoproteasome. The testis specific proteasomal subunit PSMA8, which is incorporated into the core “spermatoproteasome” (Uechi et al., 2014), responsible for the degradation of ubiquitinated proteins during spermatogenesis (Gomez et al., 2019), is known to be dysregulated in infertile patients presenting with varicocele (Agarwal et al., 2015). Further, the UPS is responsible for the removal of excess cytoplasm and ubiquitinated histones from developing germ cells (Hou and Yang, 2013). Accordingly, increased ubiquitination profiles and proteasome activity have been recorded in post-meiotic round spermatids compared to their spermatocyte precursors (Tipler et al., 1997). Downstream of testicular development there is also evidence for ubiquitination of proteins on defective spermatozoa, potentially causing a targeting of the whole cell for removal through phagocytosis by the epididymal epithelial cells prior to ejaculation (Sutovsky et al., 2001). Developmental studies in *Drosophila melanogaster* reveal a number of paralogous proteasome subunits that are testis-specific with knockouts resulting in male sterility (Belote and Zhong, 2009). Similarly, mice null for the PA200 proteasome subunit also exhibit reduced fertility (Khor et al., 2006; Qian et al., 2013).

In addition to the quality control imposed by the UPS during spermatogenesis, molecular chaperones constitute a key component of the proteostasis machinery harbored by the male reproductive system (Dun et al., 2012). In fact, a mutation in HSP90A in mice contributes to the establishment of an infertility phenotype attributed to the failure of spermatocytes to progress beyond the pachytene spermatocyte stage and the complete loss of subsequent germ cell populations (Grad et al., 2010). Interestingly, a similar phenotype is observed in HSP70 family member knockouts of both HSPA2 (Dix et al., 1996) and HSBP1 KO mice (Zhu et al., 1997; Rogon et al., 2014), with failure to produce sperm in both KO backgrounds having been linked to defects in synaptonemal complex formation and the assembly of a CDC2/cyclinB1 complex that is required for G2/M transition (Zhu et al., 1997). Following meiosis, HSPA2 also acts as a regulator of DNA packaging during spermatogenesis (Dun et al., 2012).

During testicular sperm cell development, RCS such as 4HNE and malondialdehyde (MDA) have been shown to modulate germline protein homeostasis and the stability of HSPA2. Evidence for this lies in the treatment of male germ cells with RCS, which triggers protein adduction (Bromfield et al., 2015; Nixon et al., 2019a), protein aggregation (Cafe et al., 2020) and sensitizes spermatids to demise through a ferroptotic cell death pathway (Bromfield et al., 2019). Additionally, the incubation of spermatozoa with low levels of 4HNE is known to result in an increase in proteasome activity and the subsequent proteolytic degradation of HSPA2 (Bromfield et al., 2017). A summary of our current understanding of the contribution of protein homeostasis to sperm cell development and fertility is presented in Figure 2.

In addition to the important role that chaperones play in the regulation of proteostasis in the male germline, the contribution of sirtuin proteins to fertility has also received increasing attention in recent years (Bell et al., 2014; Rato et al., 2016; Liu et al., 2017; Tatone et al., 2018; Zhang et al., 2019; Alam et al., 2020). Sirtuins (SIRTs) are a family of class III NAD-dependent deacetylases that have been extensively linked to the regulation of lifespan and the protection of cells against proteotoxicity. Seven mammalian sirtuins have been identified as residing in either the nucleus (SIRT1, 3, 6, and 7), cytoplasm (SIRT2) or mitochondria (SIRT3, 4 and 5) (Yamamoto et al., 2007). In these locations, SIRT activity is coordinated by interactions with nicotinamide adenine dinucleotide (NAD^+^). NAD^+^ is a central molecule in cellular respiration and additionally has the capacity to act as a signaling molecule and partake in redox reactions. It follows that reduced NAD^+^ availability, such as occurs during aging or in response to excessive oxidative stress, leads to a concomitant reduction in SIRT activity and contributes to the pathogenesis of disorders associated with protein misfolding (Imai and Guarente, 2016). Conversely, the deacetylation of heat shock factor 1 (HSF1) and subsequent activation of the HSR by SIRT1 (Westerheide et al., 2009) has been shown to augment protection against aggregation-based diseases. For instance, SIRT1-mediated deacetylation of tau protein, the aggregation of which is associated with the progression of AD, enhances its degradation (Herskovits and Guarente, 2013).

The deletion of sirtuin genes results in the dysregulation of male (and female) germline development (Tatone et al., 2018). As such, conditional ablation of sirtuins from male germ cells leads to reduced fertility including delayed differentiation of pre-meiotic germ cells, decreased sperm number, an increased proportion of abnormal spermatozoa (Bell et al., 2014) and disrupted acrosome biogenesis (Liu et al., 2017). These phenotypes reflect the key role that sirtuins fulfill in sensing imbalances in the metabolic state of cells and thereafter enabling the cell to mount an appropriate stress response.

### Epididymal Sperm Maturation

Although spermatozoa are morphologically mature when they exit the testis, functional maturity is not achieved until completion of epididymal transit. Owing to the purported silencing of the cell’s transcriptional and translational machinery (Ren et al., 2017), such maturational events are governed by the extrinsic factors they encounter in the epididymis and female reproductive tract (Nixon et al., 2019b). Upon leaving the testis sperm enter the proximal caput segment of the epididymis before being conveyed through the corpus to cauda (distal end) whereupon they are stored until ejaculation. Reflecting its role in the promotion of sperm maturation, defects in epididymal physiology are linked to defective sperm function and a failure to complete events related to fertilization (Yeung et al., 1998; Cooper et al., 2004). This maturation process is governed by the unique luminal microenvironment in which sperm are bathed, comprising of both soluble factors secreted by the lining epididymal epithelium, as well as those packaged into extracellular vesicles known as epididymosomes (Zhou et al., 2018).

One particular curiosity of the epididymal luminal environment is the presence of insoluble extracellular proteinaceous species resembling amyloid. Indeed, the epididymis has been shown to contain amyloidogenic prion protein both contained in epididymosomes and in soluble form (Ecroyd et al., 2004) as well as amyloid forms of the CRES (cystatin-related epididymal spermatogenic subgroup) protein, following its secretion within the proximal caput (Cornwall, 2009). Notably, the amyloid structures formed from prion and CRES proteins have been proposed to fulfill functional rather than pathological roles, including acting as scaffolds (Chau et al., 2008) and facilitating the delivery/uptake of luminal proteins into the maturing sperm cells (Cornwall et al., 2011). Together with exosomes, such interactions drive dramatic changes in the sperm proteome including the loss, gain, modification and/or repositioning of proteins. Illustrative of the scale of this phenomenon, Skerget et al. (2015) identified 732 proteins that are acquired by mouse spermatozoa and a further 1,034 proteins that are reportedly lost from these cells as they transit the epididymis. Among those proteins that display altered abundance, several proteasome subunits become enriched in maturing sperm (Skerget et al., 2015), with purported roles extending to the mediation of downstream sperm-egg interactions. While this dramatic remodeling is thought to happen in the relative absence of protein turnover, upon interrogation using stable isotope incorporation and mass spectrometry, a subset of epididymal sperm proteins (and seminal vesicle proteins) were found to be subject to rapid turnover suggesting there may be some capacity for protein production (Claydon et al., 2012). In Infertile men, alterations in the gene expression profile of epididymal epithelial cells has been observed; with those encoding proteins involved in protein folding, proteolysis, and ubiquitination, all found to be downregulated (Dube et al., 2008; Cornwall, 2009). Similarly, a conserved decrease in CRES expression has been reported in infertile mouse models generated by either ablation of the c-ros tyrosine kinase receptor or the transgenic expression of the glutathione peroxidase 5 gene (GPX5) in all the epididymal segments (Cooper et al., 2003, 2004).

In line with the identification of protein aggregates within the epididymis, this specialized tissue contains functional elements of the UPS (Baska et al., 2008), which may facilitate the removal of poor quality sperm and collaborate with extracellular chaperones to maintain proteostasis within the luminal environment. Notably, the bulk of the epididymal extracellular chaperone activity appears to rest with the glycoprotein clusterin (Humphreys et al., 1999), which is abundantly secreted into the proximal epididymal segments to account for as much as 41% of all luminal protein content (Dacheux and Dacheux, 2002). Consistent with its identification among protein aggregates in pathological disease states including AD and diabetes (Wyatt et al., 2013; Foster et al., 2019), clusterin also associates with prion protein in the epididymal lumen (Ecroyd et al., 2004). Taken together, these data suggest that clusterin may fine-tune the solubility of this protein within the luminal environment (Cornwall et al., 2011). However, whether such roles extend to regulating the extent of amyloid formation, and hence function, among aggregation-prone proteins such as CRES, remains to be investigated.

### Mature Spermatozoa

The importance of proteostasis is not relinquished once the mature spermatozoon is formed, instead the proteostasis network remains crucial for fertilization processes. As such, a myriad of barriers exist to ensure that the “fittest”/best quality sperm cells are preferentially primed for fertilization. Among these quality control measures, it has been shown that, in addition to their role in promoting sperm maturation, epididymosomes may deliver ubiquitin to moribund or poor quality spermatozoa, effectively tagging them for degradation prior to ejaculation (Sutovsky et al., 2001; Baska et al., 2008; Cornwall, 2009). Similarly, proteasome activity also appears to play a role during capacitation and acrosomal exocytosis, the final stages of sperm maturation that occur in the female reproductive tract. Thus, chemical inhibition of the sperm proteasome results in capacitation failure (Morales et al., 2003), an inability of sperm to digest the zona pellucida surrounding the oocyte (Sutovsky et al., 2004; Zimmerman et al., 2011), and an attendant decrease in fertilization rates *in vitro* (Pasten et al., 2005). In accordance with their purported role in sperm-zona interactions, a complex consisting of multiple proteasome subunits has been isolated from the surface of human sperm (Redgrove et al., 2011). Moreover, in boar spermatozoa it has been demonstrated that proteasome co-purifying proteins, such as acrosin binding protein, appear to be processed by the proteasome in a capacitation-dependent manner (Zigo et al., 2019). Furthermore, decreased proteasome activity has been correlated with decreased motility and normal morphology in human spermatozoa (Rosales et al., 2011).

The chaperoning function of heat shock proteins has also been implicated in the regulation of gamete interactions. In one of the most well studied examples, HSPA2 has been implicated in the assembly and presentation of zona pellucida receptor complexes on the surface of mature human sperm cells (Redgrove et al., 2012). In our laboratory, we have shown that the dysregulation of HSPA2 that results from 4HNE adduction under conditions of oxidative stress compromises human sperm-zona interactions and leads to a loss of fertilization potential (Bromfield et al., 2017). The physiological implications of HSPA2 dysregulation are also evident in the under-representation of this protein witnessed in the spermatozoa of infertile men (Cedenho et al., 2006; Redgrove et al., 2012). Additionally, the loss or inhibition of alternative chaperones, such as HSP90A, within the sperm proteome can also result in an infertility phenotype. Specifically, Li K. et al. (2014) have demonstrated that the pharmacological inhibition of HSP90 can prevent Ca^2+^ signaling and hyperactivation; raising the prospect that this chaperone is important for calcium homeostasis associated with sperm capacitation.

### Contribution of Somatic Cells to Germline Proteostasis

Sertoli cells are known as the “nurse” cells of the testis and, through structural support and secretory action, are responsible for initiating and regulating spermatogenesis. While their role in the maintenance of testicular proteostasis is largely unknown, analysis of testicular morphology in older men has revealed a reduction in Sertoli cell number and an increased nucleus size within the remaining Sertoli cells, which may be indicative of increased protein turnover (Pohl et al., 2019). Sertoli cells have been shown to contain multilamellar bodies with lysosome-like characteristics termed myelinosomes (Yefimova et al., 2016). Common in lysosomal storage and protein aggregation disorders, myelinosomes act as storage organelles for misfolded proteins and help maintain cellular homeostasis through non-catabolic pathways. Interestingly, myelinosomes have also been identified in Sertoli cells and seminal plasma (Yefimova et al., 2016, 2019, 2020). Huntingtin protein is present at equivalent levels in both the brain and testis, with mutations leading to both neurodegeneration and sterility (late onset) through a loss in post-meiotic cell types and testicular atrophy (Van Raamsdonk et al., 2006, 2007). Although, both pathologies can occur concomitantly at ages >39 years, no aggregated forms of mHtt have thus far been confirmed in the testes of individuals (or in mouse models) affected by HD. This remarkable difference between the brain and the testis was found to be due to myelinosomes within Sertoli cells and their unique capacity to secrete misfolded proteins to avoid aggregation and maintain cell proteostasis. The ability to release the aggregation-prone mutant form of Huntingtin (and also CFTR) but retain the normal Huntingtin protein form is facilitated by the multivesicular bodies (MVBs), whereby inhibition of MVB excretion resulted in the retention of misfolded Huntingtin inside TM4 Sertoli cells (Yefimova et al., 2016, 2019). This represents a newly characterized and remarkable form of germ cell protection afforded by its somatic cell neighbors.

### Pathological vs. Functional Protein Aggregation in the Male Germline

The formation of amyloid is not always a toxic event for cells and organisms, as such the stability and resistance provided by the cross-beta sheet structure has been exploited in essentially all phyla, appearing in cellular materials as diverse as biofilms, silk, and melanosomes (Stefani, 2004; Fowler et al., 2007; Maury, 2009; Pham et al., 2014). Functional amyloid, defined as naturally forming filamentous aggregates, have also been characterized in the context of reproductive tissues and fluid including as key structural components of the acrosomal matrix (Cornwall, 2009), epididymal plasma and the zona pellucida surrounding the oocyte (Egge et al., 2015). It follows that the structural remodeling of amyloids are linked to changes in gamete function, as illustrated by the pH-dependent disassembly of the acrosomal matrix that accompanies the acrosome reaction/release of acrosomal contents in capacitated mouse spermatozoa (Guyonnet et al., 2014). Proteomic analysis of the “amyloid core” of the mouse sperm acrosome has revealed the presence of the cystatins, CRES and cystatin C (Guyonnet et al., 2014). Mice lacking CRES display fertility defects pertaining to the inability of sperm to undergo acrosomal exocytosis *in vitro* (Chau and Cornwall, 2011). Notably, CRES proteins have also been confirmed to co-localize with positively stained amyloid in epididymal lumen (Whelly et al., 2012, 2016) and have been isolated from epididymal plasma using protein aggregation pulldown strategies (Whelly et al., 2016). Consistent with the antimicrobial properties that have been attributed to amyloid formation in other systems (Kagan et al., 2012), the potential for epididymal CRES proteins to confer antimicrobial properties and protect sperm from ascending pathogens has also recently been postulated (Hewetson et al., 2017).

Aside from CRES, cystatin C also co-localizes with amyloid structures in the epididymal fluid and with AD-associated amyloid plaques. A point mutation in human cystatin C (L68Q) triggers cystatin C to become highly unstable and aggregation-prone (Calero et al., 2001) to the point that its’ deposition in the affected individual is fatal; findings that substantiate the potential of functional amyloids to become pathological under some conditions (Whelly et al., 2014). Similarly, transgenic mice carrying the L68Q mutation of human cystatin C display reduced fertility associated with a reduction in sperm-zona pellucida binding, increased sperm cell death and agglutination. Furthermore, incubation of wild-type sperm with epididymal fluid from L68Q mutant mice recapitulates the mutant phenotype (Whelly et al., 2014).

Building on the findings of mouse models, two classes of amyloids with divergent primary sequence, but similar biochemical properties, have been isolated from human semen, namely: prostatic acid phosphatase peptides (PAP, also known as Semen-derived Enhancer of Viral Infection or SEVI (Munch et al., 2007; Lee and Ramamoorthy, 2018)) and semenogelin (SEM) amyloid (Roan et al., 2017). Both SEVI (Munch et al., 2007; Rocker et al., 2018) and SEM (Roan et al., 2011) have been associated with increased HIV transmission *in vitro* due to amyloid-enhancement of virion attachment and fusion. Contrasting to the roles of semen amyloid in HIV infection, SEM fibrils are also proposed to promote the removal of apoptotic/dysfunctional or excess sperm through engagement of macrophage clearance (Roan et al., 2017). A mouse model of *Neisseria gonorrhoeae* exposure also indicates that seminal fluid amyloid is capable of preventing infection through increased phagocytosis and stimulation of the immune response (Shamri et al., 2013). With further research into extracellular protein aggregation, the formation of amyloid in fluids of the male reproductive tract may prove to widely contribute to male fertility.

## Protein Health During Reproductive Aging in Women

Unlike the prolonged period of fertility in men, the reproductive capacity of women begins to decline precipitously in the third decade of life (Silber et al., 2017). Corollary to this, extensive efforts have been made toward defining the mechanisms underpinning the age-dependent reduction in oocyte quality/competence and the diminishing ovarian reserve. This focus has led to a compelling new understanding of the role of meiotic errors, DNA damage and chromosome abnormalities in oocyte aging and early embryo development, areas that have recently been reviewed (Greaney et al., 2018; Winship et al., 2018; Martin et al., 2019).

For reproductive cells, errors in the segregation of the chromosomes have particularly severe consequences as they give rise to aneuploid embryos that often result in miscarriage or suffer congenital abnormalities (Nagaoka et al., 2012; Greaney et al., 2018; Gruhn et al., 2019; Hassold et al., 2020). Meiosis in human oocytes is especially error prone with aneuploidy rates ranging from 15% in younger women to a staggering 30–70% in oocytes from older women and, by comparison, spermatozoa have an average aneuploidy rate of 1–4% (Nagaoka et al., 2012; Hou et al., 2013; Ottolini et al., 2015; Greaney et al., 2018; Gruhn et al., 2019). While elegant studies of DNA damage, spindle abnormalities and meiotic errors have provided unique insight into oocyte aging and meiotic failure (Mihajlović and FitzHarris, 2018; Gruhn et al., 2019; Zielinska et al., 2019; Hassold et al., 2020; Mikwar et al., 2020), advances in the application of proteomic and RNA-sequencing technology to oocytes are beginning to reveal an additional layer of complexity to this process whereby declining proteostasis contributes widely to maternal aging processes (Duncan et al., 2017). Our improved understanding of reproductive failure has been marked by the discovery that proteins critical for meiosis, such as those comprising the cohesion complex, are in fact extremely long-lived with no capacity for renewal during oocyte development (Revenkova et al., 2010; Tachibana-Konwalski et al., 2010; Burkhardt et al., 2016; Lee, 2020). Furthermore, evidence for deleterious PTMs of proteasome subunits coupled with declining proteasome activity in aged oocytes has given rise to novel streams of research into the maintenance of oocyte proteostasis (Mihalas et al., 2018). Herein, we discuss the latest findings on age-related changes to the oocyte proteome, the susceptibility of the mammalian oocyte to declining proteostasis and highlight the merit of a whole ovary approach toward a definitive understanding of reproductive aging in women.

### The Contribution of Declining Proteostasis to Oocyte Aging

The female gamete is a long-lived cell that experiences periods of prolonged arrest (of up to decades) and phases of transcriptional quiescence that place immense pressure on protein networks for cell survival. In humans, folliculogenesis commences *in utero* with the recruitment of pre-granulosa cells to germ cells to form the primordial follicle. Primordial follicles are meiotically arrested here in an extended prophase I, termed the germinal vesicle (GV) arrest (Gosden and Lee, 2010). The majority of oocytes in the ovary exist as primordial follicles constituting the ovarian reservoir. These oocytes are then sequentially recruited to the developing follicle pool at periodic intervals throughout adult life where they undergo a protracted period of maturation and eventually, ovulation (Coticchio et al., 2015). In congruence with the length of time that they are held in meiotic prophase in the ovary, these oocytes accumulate a legacy of exposure to both exogenous and endogenous insults that can lead to DNA and protein damage (Titus et al., 2013; Mihalas et al., 2017).

In understanding the susceptibility of oocytes to changes in proteostasis, it is important to note that transcription ceases when oocytes complete their growth phase prior to reentry into meiosis (Gosden and Lee, 2010; Meneau et al., 2020). During the growth phase, mRNA is synthesized for either immediate translation or storage in repressive complexes (Huarte et al., 1992; Meneau et al., 2020). During the subsequent maturation phase, these repressive complexes are removed and translation is activated (Martin et al., 2019; Meneau et al., 2020; Yang et al., 2020). At this stage the oocyte is one of the most translationally active cells in the body, placing unique pressure on both ribosome biogenesis and accurate protein synthesis to attain competence for fertilization (Gosden and Lee, 2010; Duncan et al., 2017). While the progression through meiosis is dependent upon active translation and protein accumulation (Meneau et al., 2020; Yang et al., 2020), PTMs to existing proteins also play an important role in the final stages of meiosis (Gosden and Lee, 2010; Meneau et al., 2020). This phase represents a critical window for proteostasis (from re-entry into meiosis until embryonic cleavage) whereby phosphorylation changes driven by cell cycle kinases are essential to enact meiotic resumption, maintain metaphase II arrest, and guide early preimplantation development (Kikuchi et al., 2002; Gosden and Lee, 2010; Meneau et al., 2020). Given this, it is not surprising that follicles from older mice, which have less robust protein quality control mechanisms than their younger counterparts (Duncan et al., 2017), suffer severe consequences from any loss of proteostasis during development.

Analogous to the role of Sertoli cells in the support of male germ cell development, the maturing oocytes within the preovulatory follicle, and immediately after ovulation, are enclosed within layers of nurturing cumulus cells (McLaughlin and McIver, 2009), which supply most of the metabolites and signaling molecules essential for oocyte function (Russell et al., 2016; Baena and Terasaki, 2019). While our understanding of the effects of aging on the relationship between cumulus cells and the oocyte is minimal, RNA sequencing of growing mouse ovarian follicles has revealed a marked age-dependent upregulation of oocyte specific genes that encode paracrine factors (namely *Fgf8*, *Gdf9*, and *Bmp15*), which stimulate metabolic cooperativity between the oocyte and granulosa cells. This finding is suggestive of a response to decreased support from the surrounding somatic cells to the oocyte with age, which may have downstream effects on oocyte proteostasis (Duncan et al., 2017). Importantly, this indicates that to understand changes in oocyte proteostasis with age, both the cumulus cell proteome and the oocyte proteome must be considered together.

Although proteomic studies focusing on the specific contributions of the PN to oocyte aging remain to be performed, recent studies have identified a large number of mouse oocyte proteins and transcripts that are dysregulated at key age transitions (Steuerwald et al., 2007; Grøndahl et al., 2010; Schwarzer et al., 2014; Barragán et al., 2017; Duncan et al., 2017; Wang et al., 2020). Utilizing single-follicle RNA sequencing, Duncan et al. (2017) have revealed important signatures of disrupted proteostasis in the growing GV oocytes from mice of advanced reproductive age (14–17 months). Specifically, these aged oocytes were characterized by differential expression of genes related to the nucleolus, endoplasmic reticulum and protein processing, with a significant decrease in proteins involved in: HSP binding, the UPR and misfolded protein response, mannosylation, N-linked glycosylation, and ubiquitination, in the follicles of older mice compared to their younger counterparts (6–12 weeks). This suggests that young oocytes are far more robust with regards to protein quality control than older oocytes. These findings are well supported by transcriptomic and single cell RNA sequencing studies (Grøndahl et al., 2010; Barragán et al., 2017; Wang et al., 2020) that demonstrate the dramatic modulation of mammalian oocyte transcriptomes with age, with the potential for these age-related effects on transcription to result in methylation differences (Castillo-Fernandez et al., 2020). These transcriptomic resources will enhance our understanding of changes in proteostasis that contribute to aging.

**FIGURE 3:**
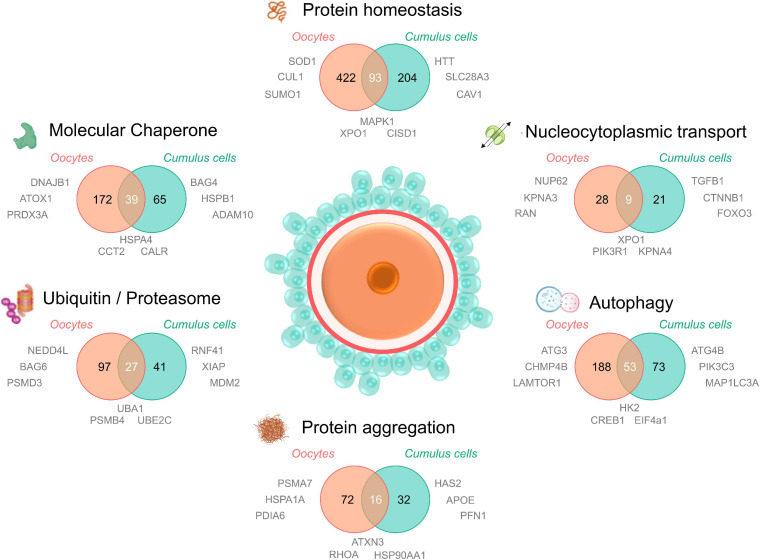
Proteomic/transcriptomic evidence for the dysregulation of the protein homeostasis network in aged oocytes and cumulus cells. To understand the contribution of proteostasis to aging in oocytes and cumulus cells, four recent proteomic and/or transcriptomic datasets of maternally aged oocytes and cumulus cells (Oocytes; Schwarzer et al., 2014; Duncan et al., 2017; Cumulus cells; McReynolds et al., 2012; Al-Edani et al., 2014) were analyzed for dysregulated elements involved in proteostasis. Proteins and transcripts were defined as “age-dysregulated” by individual cut offs (fold change and significance) specified by the authors of each primary research paper. A database of all proteostasis network related proteins was compiled from neXtProt (Zahn-Zabal et al., 2020; v2.27.0; https://www.nextprot.org; neXtProt Data release 17/01/2020) through six individual searches titled “Protein homeostasis, Protein aggregation, Autophagy, Nucleocytoplasmic transport, Ubiquitin/proteasome, and Molecular chaperone.” Human neXtProt protein accessions were then mapped to their UniProt mouse homologs to generate both mouse and human proteostasis databases (found in Supplementary File 1). Age-dysregulated proteins and transcripts listed in the Supplementary Material of the four primary manuscripts were uniformly converted to their corresponding mouse homologs and UniProt protein accessions and used to search for corresponding proteostasis related ID’s in the 6 NeXtProt database categories. These data were used to generate Venn diagrams comparing the putative age-dysregulated proteins in oocytes vs. cumulus cells and to supply relevant examples of dysregulated proteins adjacent to the component of interest. Aged oocyte IDs were from CBCF1 mice aged between 14 and 17 months (climacteric) and aged cumulus cell IDs were from cumulus-oocyte complexes from women of equivalent advanced maternal age (37–45 years). Examples included in Figure 3 were selected from age-dysregulated protein IDs. Full protein names, UniProt accessions, the proteostasis network database used to generate this figure, and lists of oocyte and cumulus cell dysregulated protein accessions can be found in Supplementary File 1. The sharing of IDs between aged oocytes and cumulus cells is putative but provides novel candidates for research into the contribution of declining proteostasis to reproductive aging. Elements of this figure were made in BioRender.

An additional resource to help understand how oocyte proteostasis changes with age is the proteomic and transcriptomic analysis performed by Schwarzer et al. (2014) on oocytes from three life stages; pubertal (3 weeks), mature (∼8 weeks), and climacteric (∼58 weeks). Interestingly, quantitative comparison of changes in the transcriptomic and proteomic molecular maps revealed a surprisingly low level of correlation (Schwarzer et al., 2014). This important finding suggests that transcriptomic analysis cannot necessarily be used as a surrogate for proteomic analysis in aged oocytes, highlighting a need for more comprehensive proteomic investigations into oocyte and ovarian aging. Using the publicly available proteomic dataset supplied by these authors (Schwarzer et al., 2014) and other relevant transcriptomic and proteomic studies of oocyte aging (McReynolds et al., 2012; Al-Edani et al., 2014; Duncan et al., 2017), we have assembled a proteostasis-centric resource to more comprehensively and rapidly survey datasets for proteostasis network proteins that are dysregulated with age (Figure 3 and Supplementary File 1). While the proteins presented in Figure 3 require experimental validation to confirm their directional changes, this *in silico* analysis sheds light on the extent of the dysregulation to proteostasis elements that occurs with age in cumulus cells and oocytes.

Notably, in aged oocytes 172 proteins related to molecular chaperones have been identified as being dysregulated, almost one quarter of which were also dysregulated in cumulus cells. Other dysregulated oocyte and cumulus cell proteins include those involved in PTMs such as SUMO1, NEDD4L, and multiple enzymes featured in ubiquitylation pathways (Supplementary File 1). Importantly, the ubiquitin encoding gene Ubb is known to be essential for meiotic progression in mouse oocytes (Ryu et al., 2008). Intriguingly, some of the dysregulated proteins mapped to karyopherin family proteins and nuclear pore proteins involved in nucleocytoplasmic transport (Figure 3). While the contribution of these proteins to oocyte aging is unknown, it is possible that, analogous to somatic cells, oocytes use nucleocytoplasmic transport to ensure subcellular management of misfolded or aggregating proteins. Recent studies of karyopherin expression has indeed mapped unique expression profiles for many of the members of this family in oocyte and follicle development (Mihalas et al., 2015), citing a possible role for these proteins in meiotic spindle function. Moreover, we have recently demonstrated that the inhibition of karyopherin A2 and B1 (KPNA2/B1) and XPO1 can exacerbate the formation of protein aggregates in male germ cells and contribute to a loss of cellular viability (Cafe et al., 2020). This provides a clear impetus to study nucleocytoplasmic transport in oocytes and cumulus cells as a potential contributing factor to oocyte aging.

Finally, several proteasomal degradation related proteins were also altered in oocytes and cumulus cells with age (Figure 3). This agrees with our findings that proteasome activity declines in aged mouse GV oocytes, which can be modeled *in vitro* through the induction of oxidative stress using 4HNE (Mihalas et al., 2018). Through this model we have also identified several core proteasome subunits that are sensitive to direct modification by 4HNE, a phenomenon that also disrupts proteostasis in somatic cells (Jung et al., 2014). 4HNE also elicits a reduction in kinetochore-microtubule and tubulin polymerization that may contribute to oocyte aging and increases in aneuploidy (Mihalas et al., 2017, 2018). Coupled with the knowledge that cohesin and several centromere-specific histones are long-lived proteins that deteriorate with age in the absence of renewal pathways (Revenkova et al., 2010; Tachibana-Konwalski et al., 2010; Burkhardt et al., 2016; Greaney et al., 2018; Lee, 2020), proteostasis defects either driven by oxidative modifications or the collapse of protein quality control over time, form a compelling explanation to support many aspects of oocyte aging and the loss of meiotic fidelity.

### Ovarian Tissue Aging and Reproductive Disorders

While many dysregulated proteins featuring in proteomic and transcriptomic datasets are common to aged cumulus cells and aged oocytes (Figure 3), a majority of the proteins mapped in each proteostasis category are in fact unique to each specific cell type. Such findings reinforce the pressing need to thoroughly investigate both the somatic and germ cell contributions to reproductive failure in women. Beyond the follicles themselves, the ovarian stroma also experiences age related changes that contribute to declining fertility. The ovarian stroma is highly heterogeneous and consists of abundant extracellular matrix (ECM) components, fibroblasts, endothelial cells, smooth muscle cells and immune cells (Tingen et al., 2011). This extra-follicular compartment of the ovary is the microenvironment in which follicles develop and provides important signaling and structural support. Although the mechanisms remain uncertain, it is known that an age-dependent increase in stromal inflammation and fibrosis also negatively impacts the quality of the gametes (Rowley et al., 2020).

An important component of the stromal compartment is the ECM and, in particular, its ubiquitous component, hyaluronan; a linear polysaccharide with pleiotropic roles in tissue structure, cell signaling and inflammation (Rowley et al., 2020; Takasugi et al., 2020). In the context of the ovary, recent work has demonstrated that total hyaluronan content in the ovarian stroma decreases with age and is effectively “replaced” by collagen leading to dramatic changes in ovarian biomechanics pertaining to increased stiffness (Amargant et al., 2020). Importantly, these age-associated changes are conserved between the mouse and humans (Amargant et al., 2020) and may be associated with the production of oocytes with compromised morphology, poor meiotic competence, and impaired granulosa cell function (Amargant et al., 2020; Rowley et al., 2020). In pathological states or during tissue aging, it also known that the fragmentation of hyaluronan yields a low molecular weight polymer (<250 kDa) linked to inflammation, fibrotic disease (Takasugi et al., 2020). Conversely, in healthy tissues, high molecular mass hyaluronan (>1 MDa) predominates and promotes tissue hydration, homeostasis and the protection of cells from stress-induced cell cycle arrest and cell death (Takasugi et al., 2020).

These latter discoveries flowed from investigation of the naked mole-rat, the longest lived rodent with a lifespan of up to 30 years; exceeding its predicted lifespan by fivefold (Edrey et al., 2011). Uniquely, the reproductive function of female naked mole-rats also increases with age until the animals reach >20 years old (Buffenstein, 2008). In understanding the remarkable lifespan of this rodent it is important to note that the naked mole-rat produces very high molecular weight hyaluronan (>6 mKD). This high molecular weight hyaluronan greatly exceeds the length of other mammalian species and is synthesized by a form of hyaluronan synthase 2 (HAS2) that differs from that found in mice (Tian et al., 2013; Faulkes et al., 2015). Additionally, naked mole-rat proteins are better at maintaining their structure and function under conditions of oxidative stress (Perez et al., 2009; De Waal et al., 2013), which can partially be explained by high proteasomal (Rodriguez et al., 2012) and autophagic activity (Zhao et al., 2014). This implies that the handling of proteotoxic stress, rather than presence or absence of the damage itself, is likely to influence cell longevity and viability (Takasugi et al., 2020). While the mechanisms promoting hyaluronan fragmentation are not entirely understood, age-dependent increases in hyaluronan degradation enzyme (hyaluronidase) activity and ROS production have been implicated (Monslow et al., 2015). This suggests that the protection of the ovary from oxidative stress and/or the bolstering of high molecular mass hyaluronan in the ovarian stroma may provide novel avenues to prevent the deleterious changes associated with aging.

### Providing a Clean Slate for Embryogenesis

Following fertilization, proteostasis has been shown to play a remarkable role in promoting healthy zygote/embryo development as it prevents the transmission of oocyte-harbored aggregates and toxic species to the next generation. In this context, studies in the model organism, *Caenorhabditis elegans*, have demonstrated that proteostatic remodeling events are capable of eliminating protein aggregates within the oocyte. This process is initiated upon mating via the action of sperm-secreted hormones, which activate a switch (VATPase catalytic subunit) to promote lysosomal acidification, effectively preparing a “clean slate” for proteostasis in the zygote (Bohnert and Kenyon, 2017). Acidification of the lysosome under these conditions occurs in concert with altered mitochondrial activity and metabolism within the oocyte, suggesting an important link between metabolism and proteostasis (Bohnert and Kenyon, 2017). Interestingly, it has also been shown that female worms devoid of sperm fail to initiate and clear protein carbonyls, while conversely, sperm-proximal oocytes in hermaphrodites efficiently remove protein carbonyls and exhibit a marked increase in the capacity to degrade protein aggregates (Goudeau and Aguilaniu, 2010). In addition to these observations in *C. elegans*, novel findings in budding yeast suggest that the process of meiotic differentiation is capable of eliminating the cellular damage induced by aging (Unal et al., 2011; King et al., 2019). This occurs through the sequestering of nuclear senescence factors, including aggregates, away from the chromosomes during meiosis II (King et al., 2019). This remarkable process leads to the elimination of abnormal cellular components such as aggregates, extrachromosomal ribosomal DNA and abnormal nucleolar material, preventing the inheritance of these factors into the newly formed gametes in a form of “meiotic rejuvenation.” Conversely, some amyloid-like protein aggregates can play functional rather than pathological roles and are utilized by cells as important regulators of gametogenesis. This was first described for the RNA binding protein Rim4 that is required for translational repression during gametogenesis in yeast (Berchowitz et al., 2015). Whilst equivalent mechanisms of proteostatic remodeling and meiotic rejuvenation have yet to be investigated in mammalian oocytes, the detection of lysosome acidification during *Xenopus* oocyte maturation raises the prospect that at least the former may be a conserved phenomenon (Bohnert and Kenyon, 2017).

### Proteostasis and Female Reproductive Health Issues

Beyond the oocyte itself, the broader contributions of proteotoxic stress to pregnancy complications and female reproductive health issues are starting to receive considerable attention. In women with endometriosis, new evidence points to an excess of ROS in the granulosa cells of growing follicles that results in a cascade of adverse sequelae beginning with the activation of ER stress pathways and leading to granulosa cell senescence, impaired cumulus oocyte complex maturation, follicle apoptosis, metabolic disturbance in the oocytes, and ovarian fibrosis; which combined underpin endometriosis-associated infertility (Lin et al., 2020). Furthermore, preeclampsia, a leading cause of pregnancy-associated morbidity and mortality, is now being viewed as a protein misfolding disorder as toxic depositions of misfolded proteins have been found to accumulate in body fluids and in the placenta of preeclamptic women (Cater et al., 2019). Concerningly, these protein aggregates are thought to contribute to the defective trophoblast invasion, placental ischemia and ER stress that are hallmark features of this pregnancy disorder (Gerasimova et al., 2019). The mechanisms behind the induction of protein misfolding in preeclamptic pregnancies are undoubtedly complex, however, the novel discovery of the anti-aggregation chaperone activity of pregnancy zone protein (PZP), a protein that is dramatically increased in maternal blood plasma during pregnancy, may aid our understanding of this phenomenon. PZP has been shown to efficiently inhibit *in vitro* aggregation of several vulnerable proteins (Cater et al., 2019). Thus, PZP upregulation during pregnancy may represent an important maternal adaptation to maintain extracellular proteostasis during gestation and it is possible that it is the disruption, or overwhelming of PZP during pregnancy that underpins the accumulation of misfolded proteins (Cater et al., 2019). Further research will need to be conducted to determine whether the stabilization of PZP throughout pregnancy can reduce the incidence of preeclamptic pregnancies. Given increasing evidence that women who have experienced preeclampsia may be at an increased risk of adverse outcomes in other protein misfolding disorders such as AD (Theilen et al., 2016) and cataract formation later in life (Auger et al., 2017), it will be important to investigate the mechanistic commonalities between germ and somatic cell protein homeostasis.

## Discussion and Conclusion

### Organismal Proteostasis and the Compromise of Lifespan for Reproduction

Reproduction is a high-cost scenario and, with current reproductive success being preponderant to future reproduction, natural selection often favors genes that mediate survival earlier in life as opposed to after the peak of reproductive activity (Maklakov and Immler, 2016). This causal, inverse relationship between reproduction and lifespan is perhaps best surmised in the “disposable soma theory,” which posits that investment into the germline is prioritized over that of the soma (Kirkwood, 1977). Whilst empirical evidence supporting the trade-off between life span and reproduction is limited, pioneering studies in *C. elegans* and *D. melanogaster* have recently given credence to this model.

In *C. elegans*, the ability to maintain somatic cell proteostasis declines dramatically upon the initiation of reproduction (Khodakarami et al., 2015). Remarkably, germline ablation in *C. elegans* (i.e., removal of the germline without an attendant removal of the whole gonad), promotes longevity by triggering a signaling network regulated by both transcription factors and microRNAs (Ermolaeva et al., 2013; Shemesh et al., 2013). This response converges on the FOXO transcription factor, DAF-16, which activates downstream proteasome-related genes, increasing stress resistance and ultimately organismal longevity (Vilchez et al., 2012; Shemesh et al., 2013). Strikingly, *mir-71* expression in neuronal cells is also enough to rescue gonadal longevity, pointing to a cell-non-autonomous relationship between the nervous system and the gonad, a prime example of the importance of organismal proteostasis (Antebi, 2013). Similarly, interconnections between stress response pathways, the reproductive system and metabolism have been linked to the expression of glucagon-like-peptide 1 (*glp1)*. Thus, stress resistance and the maintenance of somatic cell proteostasis, can be promoted in germline ablation models wherein *glp1* mutations prevent germ cell proliferation prior to adulthood transition. Curiously, these mutations also facilitate the suppression of protein aggregation and polyQ disease progression (Shemesh et al., 2013). Germline-less *C. elegans* are more resistant to proteotoxic stress conditions such as heat stress and are better protected from protein aggregate and polyglutamine-driven toxicity, a phenomenon partially attributed to increased proteasome activity in the somatic cells (Vilchez et al., 2012). These cells also exhibit increased autophagic activity and lipase-4-dependent lipolysis, which may modulate the longevity phenotype through increased lipid clearance or enhanced regulation of lipid-derived signaling molecules such as aldehydes (Folick et al., 2015).

Not unlike *C. elegans*, a dysregulation of PN gene expression and a concomitant decrease in proteasomal catalytic activity have been noted during the aging process in both male and female *Drosophila*. By contrast, proteasome activity is retained within the germ cells of these flies (Tsakiri et al., 2013), with molecular chaperones and proteasome machinery normally elevated 2-8-fold in the abdomen compared to the thorax (Fredriksson et al., 2012)- further highlighting the battle of resource allocation between the germ line and the soma. These unique molecular links between reproduction and lifespan form a growing body of literature on organismal proteostasis that is currently being verified in mammalian model species. Importantly, these findings highlight the importance of the maintenance of proteostasis in the germline for the health and wellbeing of organisms as the remodeling of the organismal stress response associated with the onset of reproduction may render organisms more susceptible to environmental insults. Thus, in depth studies of the mammalian proteostasis network and mechanisms for aggregate clearance are needed to better understand male and female reproductive health throughout life.

### Therapeutics Targeted Toward Proteostasis to Promote Reproductive Health

In consideration of the wealth of reproductive and non-reproductive disorders underpinned by protein misfolding and aggregation, it is apparent that there are substantial dividends to be gained from modulation of the proteostasis network in the context of protecting neuronal and germ cells alike. In evaluating appropriate strategies, the framework provided by the FoldEx model is particularly informative (Powers et al., 2009). That is, effective amelioration of a protein-misfolding disorder necessitates that a misfolded protein must shift to within the proteostasis boundary delineated by the stability, misfolding rate and folding rate of the protein. Taking pharmacological chaperones as an example, there are three theoretical ways in which loss-of-function misfolding diseases could be modulated using these reagents. In principle, pharmacological chaperones could function by either increasing the folding rate of a protein, thermodynamic stabilization or decreasing misfolding rate by stabilization of native protein structure; the latter two of which have been experimentally verified (Powers et al., 2009). Thus, proteostasis regulators could be used to alter the composition and concentration of proteostasis network components and hence move the proteostasis boundary, potentially mimicking UPR and HSR including the induction of HSP70 and HSP90 families of chaperones (Westerheide and Morimoto, 2005) and modulation of histone deacetylases (HDACs) (Gan, 2007; Outeiro et al., 2007; Westerheide et al., 2009).

Illustrative of this potential, HDAC modulation through class II histone deacetylases (Sirtuins), has gained considerable interest in the field of neurodegeneration owing to the potential to ameliorate stress responses and protein aggregation and promote healthy aging (Merksamer et al., 2013). Importantly, a role for SIRT proteins in fertility is beginning to emerge following several reports of a defective reproductive phenotype in SIRT1-null animals (McBurney et al., 2003; Coussens et al., 2008). The loss of SIRT1 in both sexes results in infertility phenotypes, characterized in the male by severely disrupted spermatogenesis, reduced spermatogonial stem cell population and DNA damage (Coussens et al., 2008). Despite the promise of SIRT1 activators to reduce stress sensitivity in other tissues, the ability of SIRT1 to protect male germ cells against oxidative stress has yet to be evaluated. Nevertheless, we have recently begun to explore whether SIRT1 activation can modulate levels of protein aggregation in developing male germ cells. Intriguingly, the use of the SIRT1 activator, SRT1720, resulted in a significant decrease in protein aggregation in both pre- and post-meiotic populations of pachytene spermatocytes and round spermatids (Cafe et al., 2020). Moreover, with regards to female fertility the downregulation of SIRT1 is associated with reduction of the ovarian reserve and important roles for both SIRT1 and SIRT3 in the sensing of redox state and energy homeostasis have been reported in oocytes, granulosa cells and early embryos (Tatone et al., 2015, 2018). Indeed, SIRT1 has now been shown to slow the age-related decline in oocyte quality thereby sustaining female fertility (Iljas et al., 2020). However, SIRT3 appears to be dispensable for female fertility (Iljas and Homer, 2020). Several of the SIRT family enzymes are yet to be explicitly explored in relation to reproductive health and this will likely be an important avenue to pursue in relation to the maintenance of proteostasis during reproductive aging in men and women. In addition to the direct modulation of Sirtuins, several recent studies have demonstrated that supplementation with the NAD+ precursor nicotinamide mononucleotide (NMN) improves the quality of maternally aged oocytes through the restoration of both mitochondrial function and meiotic competency (Wu et al., 2019; Bertoldo et al., 2020; Miao et al., 2020). These outcomes could be recapitulated in mice through the overexpression of SIRT2 (Bertoldo et al., 2020). Beyond the oocyte, NMN was also able to improve developmental competency in the embryos produced from aged animals (Bertoldo et al., 2020; Miao et al., 2020). While NMN was not able to protect the ovary from the severe damage caused by chemotherapy (Stringer et al., 2019), the bolstering of NAD+ provides promise for the improvement of reproductive outcomes of women of advanced maternal age.

In many diseases and disorders of proteostasis, recent progress toward therapeutics has been enabled through the targeting of the integrated stress response (ISR). The ISR is an evolutionarily conserved signaling network coupled to the UPR and HSR, operating through the reprogramming of translation (Costa-Mattioli and Walter, 2020). Importantly, this network modulates diverse stress inputs (such as nutrient deprivation, proteostasis defects and redox imbalance) and leads to a central output – a reduction in the translation of specific mRNAs that promote proteostasis. Evidence linking the ISR to the maintenance of cellular homeostasis and apoptosis in the reproductive organs of men, allied to its contribution to infertility in animal models (Karna et al., 2019), identifies the ISR as a potentially attractive therapeutic target. Additionally, the discovery of PZP as an anti-aggregation factor for proteins involved in preeclampsia (Cater et al., 2019) opens an important window for the regulation of protein misfolding disorders that affect female reproductive health. Our ability to capitalize on these novel proteostasis interventions in the context of infertility research is now predicated on improvements in our fundamental knowledge of aggregation-prone proteins in gametes and the PN in germ cells more broadly.

## Author Contributions

SC, EB, and BN were responsible for study design, execution, analysis, manuscript drafting, and critical discussion. HE, JM, and DS-B participated in manuscript editing and critical discussion. All authors contributed to the article and approved the submitted version.

## Conflict of Interest

The authors declare that the research was conducted in the absence of any commercial or financial relationships that could be construed as a potential conflict of interest.
