# Supplementary material for: Proteostasis in the Male and Female Germline: A New Outlook on the Maintenance of Reproductive Health
Source: Front Cell Dev Biol. 2021 Apr 16;9:660626. doi: 10.3389/fcell.2021.660626 (PMC8085359; doi:10.3389/fcell.2021.660626)
Supplement: Supplementary file 2 [file Table_1.docx]

**Table 1: Parallels between infertility and neurodegeneration**

| Contributing factors | | Male infertility | Female infertility | Protein misfolding diseases |
| --- | --- | --- | --- | --- |
| **Protein dysregulation** | - Superoxide dismutase (SOD) | Infertility; Drosophila SOD1 -/- males are infertile (Reveillaud et al. 1994; Şahin et al. 2017), SOD1 is dysregulated in oligozoospermia patients with varicocele (Agarwal, Panner Selvam, and Baskaran 2020) | SOD activity in follicular fluid from PCOS infertile women is significantly lower than in the control group (Seleem et al. 2014); SOD1 important for luteal function and the maintenance of fertility in female mice (Ho et al. 1998; Noda et al. 2012) | SOD mutation contributes to the accumulation of protein aggregates and progression of Amyotrophic Lateral Sclerosis, 20% familial form (Kumar et al. 2017) |
|  | - Protein disulfide isomerase (PDI) | Important for sperm-oocyte interaction (PDIA6) and epididymal maturation (PDIA3) (Bromfield et al. 2016) | PDIA3 plays a crucial role in sperm de-condensation in the oocyte by reducing protamine disulfide bonds (Li et al. 2014) | PDI has been identified as a risk factor for Amyotrophic Lateral Sclerosis (Kwok et al. 2013) |
|  |  |  | PDIA4 and PDIA6 associated with disrupted redox homeostasis in porcine oocytes (Yuan, Wheeler, and Krisher 2012) | Loss of PDI increases SOD aggregation and colocalizes with aggregates in Amyotrophic Lateral Sclerosis - affected brain tissue (Jaronen, Goldsteins, and Koistinaho 2014; Jeon et al. 2014) |
|  | - Heat Shock Protein (HSP) 70 family | HSPA2 loss leads to failed meiosis, apoptosis, infertility (Bromfield et al. 2017; Dix et al. 1996) and testicular cancer (Dias et al. 2020) | HSP70 expressed at stable levels throughout oocyte aging and no female infertility phenotype present in *Hspa2*-/- mice (Dix et al. 1996). However, HSF1-/- oocytes have delayed germinal vesicle breakdown phenotype and meiotic arrest and abnormal pronuclei formation (Le Masson et al. 2011) | HSPA2 colocalizes with aggregate deposits. Parkinson’s Disease linked to HSPA9 mutation (Smith, Li, and Cheetham 2015) |
|  |  | HSPA2 is reduced in sperm of oligozoospermic patients (Cedenho et al. 2006) |  | α-synuclein aggregation in PD decreased by HSP70 (Klucken et al. 2004; Smith, Li, and Cheetham 2015; Campanella et al. 2018) |
|  |  | HSPA2 is dsyregulated in varicocele and asthenozoospermia (Agarwal, Panner Selvam, and Baskaran 2020) |  |  |
|  |  | HSPA5 loss leads to oligozoospermia and HSPA1L mutations cause male infertility (Kohan, Tabiee, and Sepahi 2019) |  |  |
|  | - Chaperonin (TRiC and CCT) | CCT complex is critical for both spermatogenesis and oocyte interactions (Dun et al. 2011) and is reduced in sperm from infertile donors (Samanta et al. 2019) | CCT2, CCT3 and CCT5 are decreased in aged MII oocytes (Hamatani et al. 2004) and CCT2 is decreased in GV oocytes (Pan et al. 2008) | Knockdown of TRiC enhances the aggregation of mutant Huntington protein (Shen 2013) |
|  | - Cystic fibrosis transmembrane conductance regulator | Mutations in CFTR cause decreased secretion and epididymal regression (Fraser-Pitt and O'Neil 2015; Hamada, Esteves, and Agarwal 2013) | CFTR regulates fluid volumes and HCO₃- secretion in the uterus and oviduct with functional implications for sperm transport and blastocyst implantation (Chan et al. 2009) | CFTR mutation causes protein misfolding and degradation, leading to Cystic Fibrosis (Fraser-Pitt and O'Neil 2015) |
| **Oxidative stress** | | By-products can form deleterious protein adducts, resulting in misfolding/loss of function e.g. 4HNE adducted HSPA2 contributes to failed oocyte interactions (Bromfield et al. 2017) | Reactive carbonyl species such as 4HNE modify several proteasome subunits and cause loss of proteasome activity in GV mouse oocytes (Mihalas et al. 2018) | Oxidative adduction of proteins leads to formation of aggregates in Parkinson’s Disease, Alzheimer’s Disease and Amyotrophic Lateral Sclerosis (Lee, Giordano, and Zhang 2012) |
|  |  |  | Oxidative stress decreases mitochondrial membrane potential in oocytes. Postovulatory aging underpinned by oxidative stress, this can be alleviated with SIRT1 activator Melatonin (Lord et al. 2013) |  |
|  |  | Oxidative stress is commonly elevated in infertile male patients (Tremellen 2008) | Conditions affecting fertility in women, including endometriosis, polycystic ovary syndrome and recurrent pregnancy loss, are associated with a systemic increase in reactive oxygen species (Gupta et al. 2013) | 4HNE levels increase in neurodegenerative conditions (Yoritaka et al. 1996) and is used as a biomarker of PD (Popa-Wagner et al. 2013) and 4HNE increases amyloid-β toxicity through increased oligomer production (Siegel et al. 2007) |
| **Ageing** | | Decreased semen quality parameters, including reduced sperm count, morphology and motility (Ford et al. 2000; Frattarelli et al. 2008) | Increased DNA damage and mutational load, high rates of aneuploidy, increased miscarriage and declining oocyte proteostasis (Duncan et al. 2017; Greaney, Wei, and Homer 2018) | Identified as single biggest risk factor for neurodegeneration, linked to decreased proteasome activity, reduced proteostasis network kinetics, and increased protein aggregation (Hartl and Hayer-Hartl 2002) |
|  |  | Manifests in elevated risk of offspring mutational load (Drevet and Aitken 2020) | Decreased oocyte quality and quantity with age (Winship et al. 2018) |  |
| **Failed proteasomal degradation** | | 4HNE dysregulates proteasome activity in male germ cells leading to aberrant degradation of HSPA2 and correlating with lower overall sperm quality (Rosales et al. 2011) | Several proteasome subunits dysregulated at proteomic and transcriptomic level in aging oocytes and cumulus cells (Duncan et al. 2017; McReynolds et al. 2012; Mihalas et al. 2017) | Cross-linked proteins inhibit the proteasome, preventing degradation and perpetuating aggregate accumulation (Hohn, Konig, and Grune 2013) |
|  |  | Loss of proteasome regulatory subunit (PA200) results in spermatocyte arrest (Khor et al. 2006) | 4HNE adduction of proteasome subunits and loss of proteasome activity in aged oocytes (Mihalas et al. 2018) | Proteasome activity is decreased in brain regions corresponding to neuron degeneration in patients with AD (Keller, Hanni, and Markesbery 2000) |
|  |  | PSMA8 dysregulated in infertile patients with varicocele (Agarwal et al. 2015) |  |  |
|  |  | Histone retention due to failed proteasomal degradation contributes to poor sperm morphology (Wang et al. 2019) |  |  |
| **Autophagic-lysosomal dysregulation** | | Loss of Atg7 results in infertility due to failed acrosome biogenesis contributing to a globozoospermia-like phenotype (Wang et al. 2014) | Autophagy is important for establishing primordial follicle pool. Atg7-/- display 50% reduction in oocytes during neonatal period in mice and can lead to Premature Ovarian Insufficiency (POI) (Song et al. 2015; Sun et al. 2018) | Dysregulated in ALS through mutations in SQSTM1/P62 and TDP43 (Donde et al. 2020; Ramesh and Pandey 2017) |
|  |  | Atg7-/- results in manchette deformation (Shang et al. 2016) | BECN1 and ATG7 are essential for the maintenance of the ovarian reserve of primordial follicles (Gawriluk et al. 2011; Peters et al. 2020) | sqstm1-/-, Atg7-/- and Atg5-/- mice show high levels of neurodegeneration and increased deposits of ubiquitinated protein aggregates (Hale et al. 2013) |
|  |  | Autophagy is increased in spermatozoa from men with cryptorchidism (Yefimova et al. 2019) | LC3B abundant in granulosa cells, cumulus cells, and oocytes during atresia of pig antral follicles (Gioia et al. 2019) | Loss of ATG7 and ATG5 sensitise neurons to ROS (Lee, Giordano, and Zhang 2012) |
|  |  |  |  | Neurodegenerative tissue displays a downregulation of Beclin 1 (Pickford et al. 2008) |
|  |  |  |  | Degradation of Huntington protein requires autophagy (Qi et al. 2012) |
| **Sirtuins** | | *Sirt1* germ cell-conditional KO results in failed acrosome development, disrupted autophagy in spermatids and decreased motility parameters (Liu et al. 2017) | *Sirt1 -/-* females have reduced fertility, ovulation defects and delayed reproductive maturity (Tatone et al. 2018; Tatone et al. 2015) | SIRT1 enhances neuronal survival in cell culture models of AD (Kim et al. 2007) |
|  |  |  |  | SIRT2 offers neuroprotection and decreases α-synuclein toxicity (Yalcin 2017) |
| **Karyopherin dysfunction and mislocalization of proteins** | | Karyopherins are under-represented in infertile sperm (Samanta et al. 2019) and may contribute to failed nuclear compaction | Karyopherins have distinct expression patterns in developing and mature oocytes (Mihalas et al. 2015). Importin a2 is required for normal gametogenesis and Importin a2 null female *drosophila melanogaster* are infertile (Mason et al. 2002) | Mislocalization of aggregates increases their toxicity with aggregates able to block the nuclear pore preventing correct nucleocytoplasmic transport (Eftekharzadeh et al. 2018) |
|  |  |  |  | Amyotrophic Lateral Sclerosis causes mislocalization of karyopherins (Kim and Taylor 2017) |
| **Post-translational modifications** | | PTMs are important for modulating sperm proteome as they are transcriptionally and translationally silent but are also implicated in infertility, failed fertilisation and abnormal sperm morphology (Maciel, Tamashiro, and Bertolla 2019) | Cell cycle kinases such as MAPK direct meiotic resumption, maintain metaphase II arrest in oocytes and assist in preimplantation embryo development in the absence of transcription and translation (Gosden and Lee 2010) | Hyperphosphorylation of Htt leads to nuclear accumulation and HD (Atwal et al. 2011) |
|  |  | Phosphorylation plays physiological roles in sperm maturation (Nixon and Bromfield 2018) | Inhibition of neddylation causes meiotic arrest in mouse oocytes (Yang et al. 2019) | TDP-43 phosphorylation, oxidisation and acetylation increases aggregation (Ren et al. 2014) |
|  |  | Phosphorylation, acetylation, and SUMOylation of the androgen receptor govern its subcellular localization, intermolecular associations, and DNA binding (Fu et al. 2006) | Loss of SUMO-conjugating enzyme Ube2i in oocytes results in infertility in mice (Rodriguez et al. 2019) | SUMOylation of SOD1 increases aggregation (Sambataro and Pennuto 2017) |
|  |  |  |  | Oxidative modification of proteasome subunits decreases activity (Farout et al. 2006; Hohn and Grune 2014) |
|  |  |  |  | Multiple chaperones are oxidatively modified in AD (Di Domenico et al. 2014) |
| **Pathogenic protein aggregation** | | **Unknown** | **Unknown** | Contributes to a numerous diverse range of progressive disorders including neurodegenerative diseases, diabetes and cataract formation |
